# Supplementary material for: Proteomic and structural comparison between cilia from primary ciliary dyskinesia patients with a DNAH5 defect
Source: Front Mol Biosci. 2025 Jul 17;12:1593810. doi: 10.3389/fmolb.2025.1593810 (PMC12310455; doi:10.3389/fmolb.2025.1593810)
Supplement: Supplementary file 1 [file Supplementaryfile1.docx]

# Appendix


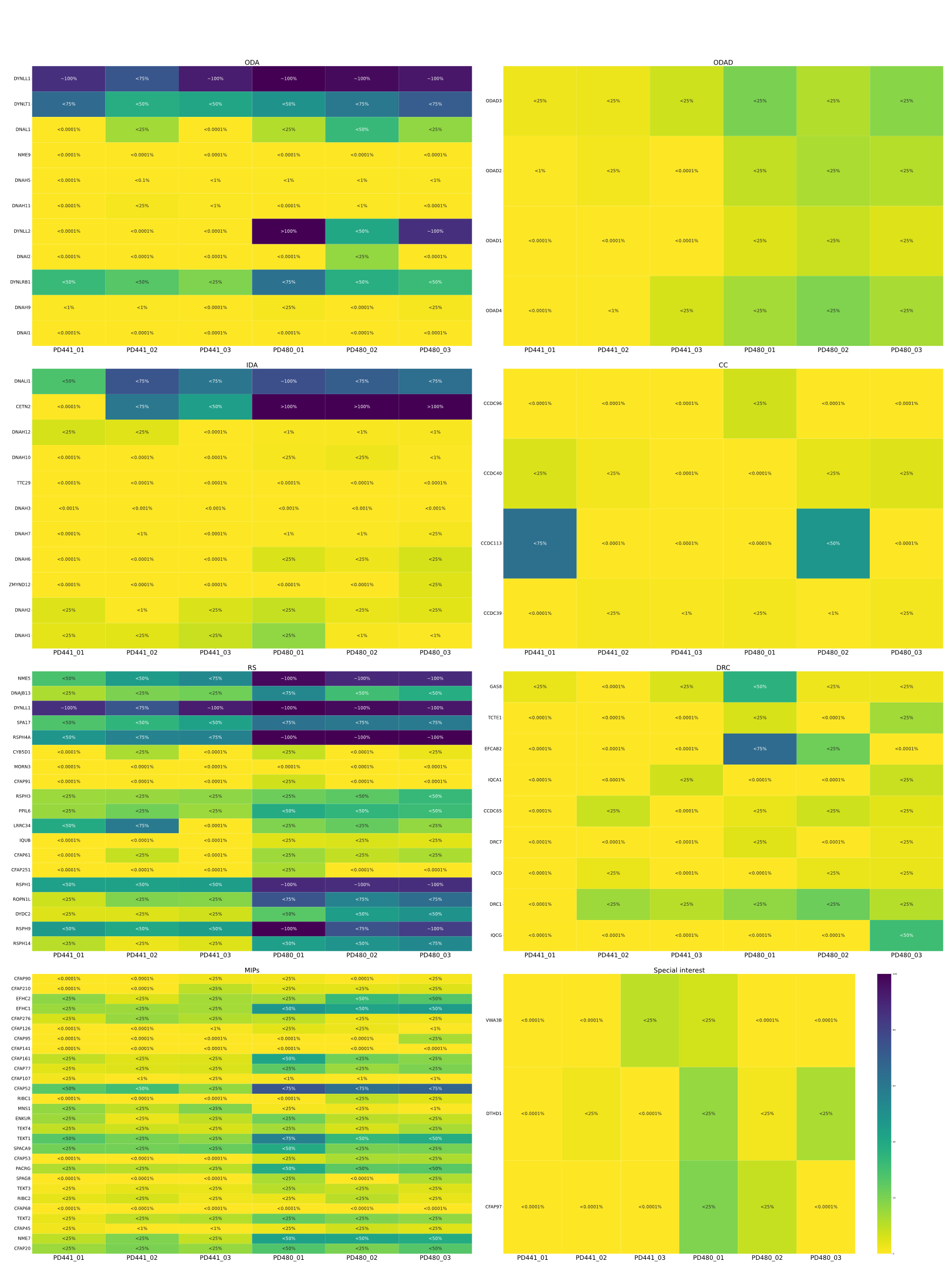


**Figure S1**. Heat maps summarizing the LC/MS-MS results for grouped ciliary proteins in PD441 and PD480 samples. Protein intensity values were normalized by the TUBB4B intensity. Values represent a percentage from wild type abundance. Percentages were binned to be <0.0001%, <0.001%, <0.01%, <0.1%, <1%, <25%, <50%, <75%, ~ 100%, and >100%.

**
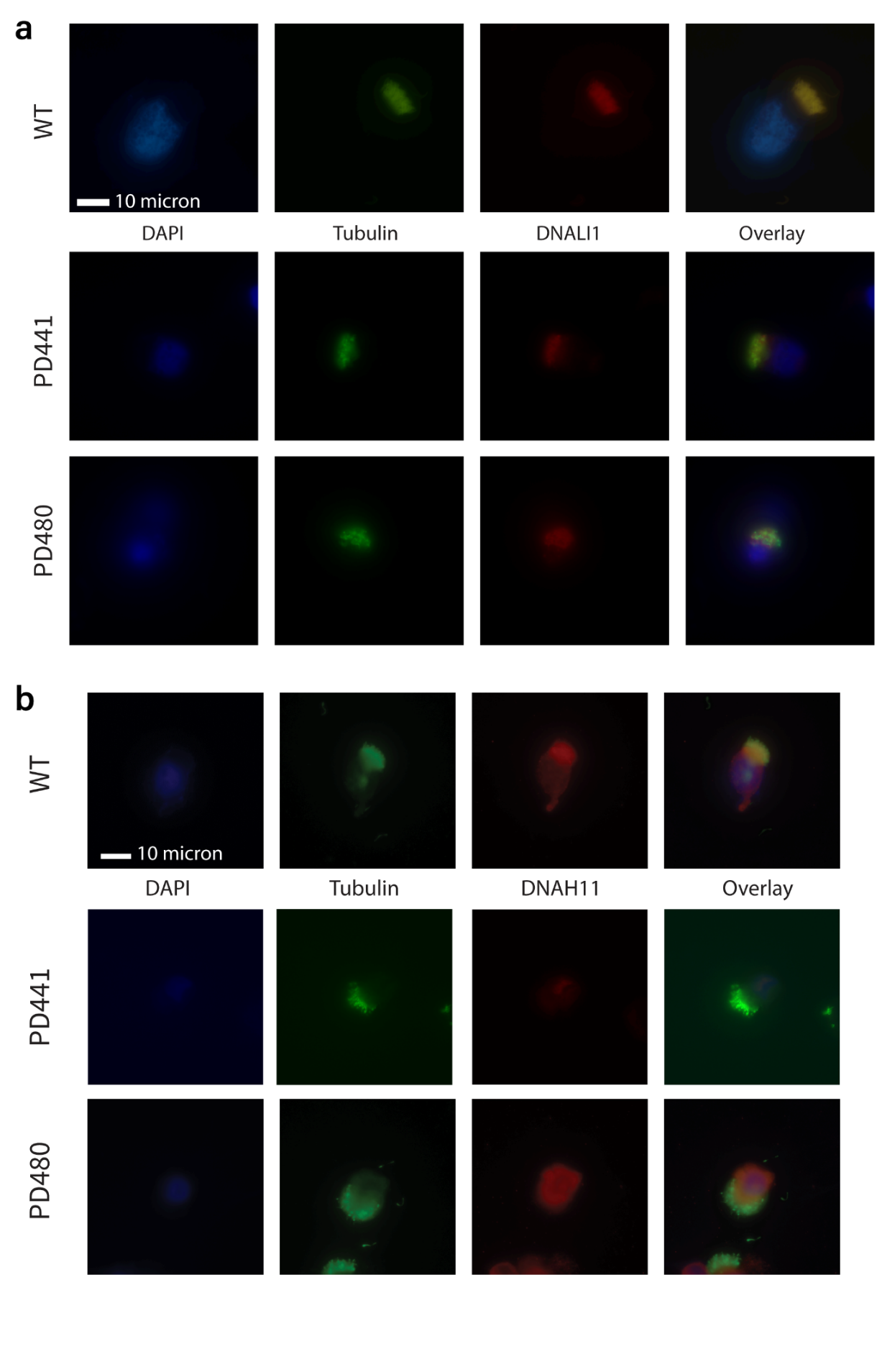
**

**Figure S2.** Immunofluorescence staining of WT and PCD patient cells PD441 and PD480 with A) DNALI1 and B) DNAH11. Acetylated tubulin is stained in green and DNA (DAPI) in blue.


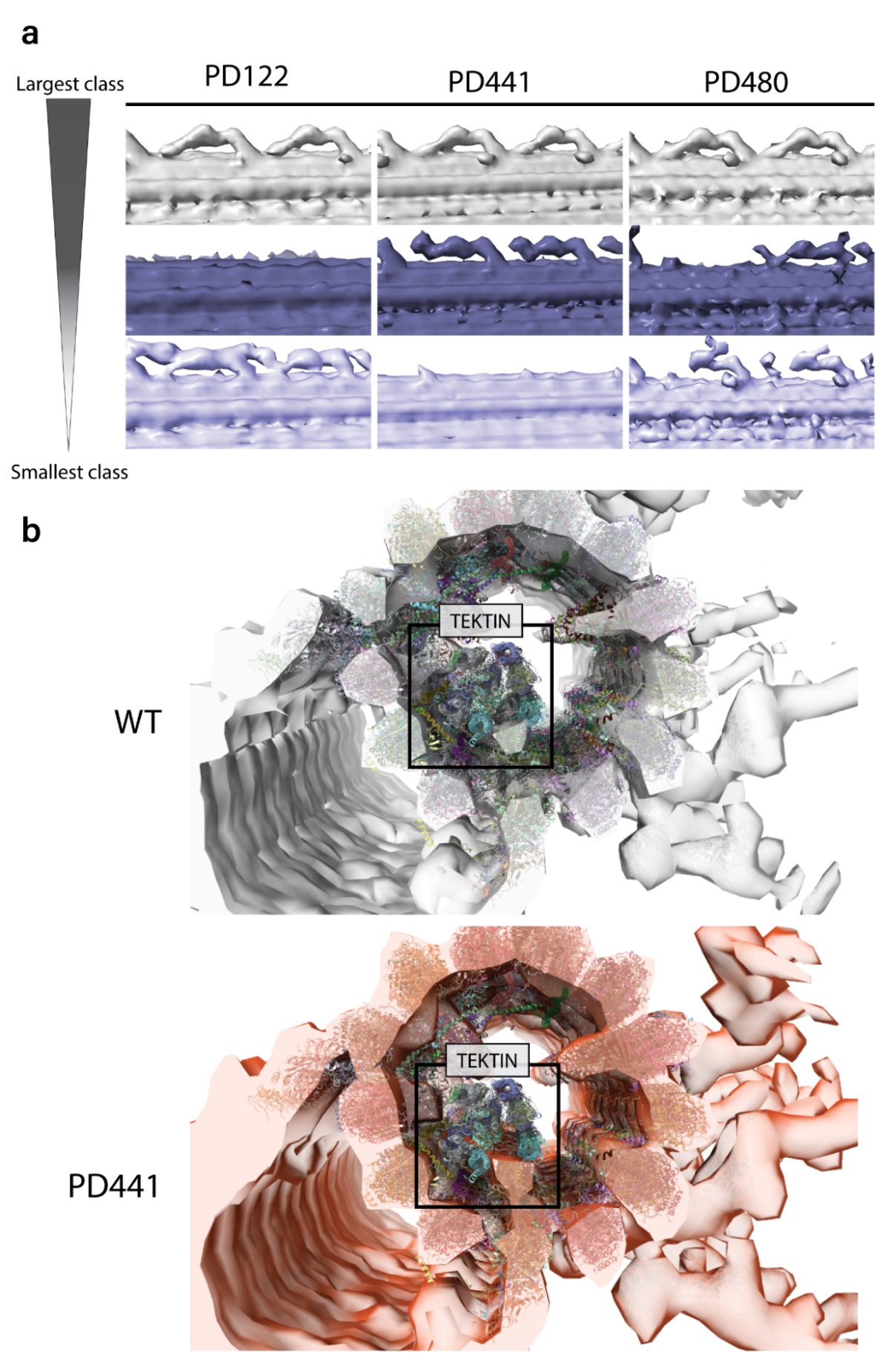


**Figure S3**. Structural analysis of PCD patient 24- and 96-nm periodic unit of the microtubule doublet (MTD) A. Classification of ODA particles for each patient. PD122 and PD441 show classes that have a complete loss of the docking complex, whereas all major PD480 classes have a visible docking complex. B. Fitting of atomic model 7RRO [Gui et al 2021] into the 96-nm modular repeat tomographic maps of WT and PD441. Images were generated in ChimeraX (Meng et al., 2023).

**Table S1**. Antibodies for IF staining

| **Protein** | **Manufacturer** | **Product number** | **Dilution** |
| --- | --- | --- | --- |
| beta-tubulin | Sigma-Aldrich | T7451 | 1:1000 |
| DNAH5 | Prestige, Sigma-Aldrich | HPA037470 | 1:100 |
| DNAH9 | Prestige, Sigma-Aldrich | HPA052641 | 1:50 |
| DNAH11 | Prestige, Sigma-Aldrich | HPA045880 | 1:50 |
| DNAI1 | Prestige, Sigma-Aldrich | HPA021649 | 1:100 |
| DNAI2 | Prestige, Sigma-Aldrich | HPA050565 | 1:100 |
| DNALI1 | Prestige, Sigma-Aldrich | HPA028305 | 1:100 |
| GAS8 | Prestige, Sigma-Aldrich | HPA041311 | 1:200 |
| RSPH1 | Prestige, Sigma-Aldrich | HPA017382 | 1:100 |
| RSPH4a | Prestige, Sigma-Aldrich | HPA031197 | 1:200 |
| RSPH9 | Novus, Bio-Techne | NBP1-86750 | 1:100 |
| goat anti-mouse | Invitrogen | A21121 | 1:2500 |
| goat anti-rabbit | Invitrogen | A11035 | 1:1000 |

| Table S2. Tomography data analysis parameters for wild type and patient data | | | |
| --- | --- | --- | --- |
| **Microscope** | | Titan Krios | |
| **Camera** | | Gatan K2 Summit | |
| **Energy filter** | | GIF Quantum LS | |
| **Magnification** | | 33000x | |
| **Voltage (kV)** | | 300 | |
| **Number of frames per tilt image** | | 10 | |
| **Total electron exposure (e-/A2)** | | 60-80 | |
| **Defocus range** | | 3-5 | |
| **Sample name** | **Number of tomograms** | **Particle number (24-nm/96-nm)** | **Resolution 96-nm structure (A)** |
| WT | 54 | 7704/499 | 35 |
| PD122 | 19 | 7408/714 | 36 |
| PD441 | 50 | 7862/959 | 36 |
| PD480 | 25 | 4391/733 | 35 |

**Table S3**. DNAH5 mutations of patient samples used in this study

| **Patient** | **DNA change** | **Protein change** |
| --- | --- | --- |
| PD122 | [13692093_13944547del] + [4348C→T] | [?] + [Q1450X] |
| PD441 | [8998C→T] + [799G→T] | [R3000X] + [V267F] |
| PD480 | [13194_13197del] + [11761+1_11762-1)_(11883+1_11884-1)del] | [D4398EfsX16 + [?] |

**Table S4**. Clinical information concerning PD122

| **PD122** | | | | |
| --- | --- | --- | --- | --- |
| Sex | female | | | |
| Age | 19 years | | | |
| Age at diagnosis | 2 years | | | |
| **Symptoms overview** | | | | |
|  | | **Yes** | **No** | **Not known** |
| Neonatal respiratory distress | | x |  |  |
| Chronic rhinosinusitis | | x |  |  |
| Hearing impairment | | x |  |  |
| Persistent otitis media | | x |  |  |
| Chronic cough | | x |  |  |
| Recurrent bronchitis/pneumonia | | x |  |  |
| Bronchiectasis | |  |  | x |
| Laterality defects | | x |  |  |
| Hydrocephalus | |  | x |  |

**Table S5**. Clinical information concerning PD441

| **PD441** | | | | |
| --- | --- | --- | --- | --- |
| Sex | female | | | |
| Age at brushing | 33 years | | | |
| Age at diagnosis | 33 years | | | |
| **Symptoms overview** | | | | |
|  | | **Yes** | **No** | **Not known** |
| Neonatal respiratory distress | | X |  |  |
| Chronic rhinosinusitis | | X |  |  |
| Hearing impairment | |  |  | x |
| Persistent otitis media | |  | X |  |
| Chronic cough | | X |  |  |
| Recurrent bronchitis/pneumonia | | X |  |  |
| Bronchiectasis | |  |  | x |
| Laterality defects | |  | X |  |
| Hydrocephalus | |  | x |  |

**Table S6**. Clinical information concerning PD480

| **PD480** | | | | |
| --- | --- | --- | --- | --- |
| Sex | Male | | | |
| Age at brushing | 27 years | | | |
| Age at diagnosis | unknown | | | |
| **Symptoms overview** | | | | |
|  | | **Yes** | **No** | **Not known** |
| Neonatal respiratory distress | |  |  | x |
| Chronic rhinosinusitis | |  |  | x |
| Hearing impairment | |  |  | x |
| Persistent otitis media | |  |  | x |
| Chronic cough | | X |  |  |
| Recurrent bronchitis/pneumonia | |  |  | x |
| Bronchiectasis | | X |  |  |
| Laterality defects | |  |  | x |
| Hydrocephalus | |  | X |  |
